# Effect of Aneurysmal Subarachnoid Hemorrhage on Word Generation

**DOI:** 10.1155/2014/610868

**Published:** 2014-03-02

**Authors:** Daniella Ladowski, Winnie Qian, Anish N. Kapadia, R. Loch Macdonald, Tom A. Schweizer

**Affiliations:** ^1^Keenan Research Centre for Biomedical Science, the Li Ka Shing Knowledge Institute, St. Michael's Hospital, 209 Victoria Street, Toronto, ON, Canada M5B 1T8; ^2^Division of Neurosurgery, Department of Surgery, Faculty of Medicine, University of Toronto, Toronto, ON, Canada M5S 1A8; ^3^Division of Neurosurgery, St. Michael's Hospital, Toronto, ON, Canada M5B 1W8; ^4^Institute of Biomaterials and Biomedical Engineering, University of Toronto, Toronto, ON, Canada M5S 3G9

## Abstract

*Background*. Aneurysmal subarachnoid hemorrhage (aSAH) survivors commonly exhibit impairment on phonemic and semantic fluency tests; however, it is unclear which of the contributing cognitive processes are compromised in aSAH patients. One method of disentangling these processes is to compare initial word production, which is a rapid, semiautomatic, frontal-executive process, and late phase word production, which is dependent on more effortful retrieval and lexical size and requires a more distributed neural network. *Methods*. Seventy-two individuals with aSAH and twenty-five control subjects were tested on a cognitive battery including the phonemic and semantic fluency task. Demographic and clinical information was also collected. *Results*. Compared to control subjects, patients with aSAH were treated by clipping and those with multiple aneurysms were impaired across the duration of the phonemic test. Among patients treated by coiling, those with anterior communicating artery aneurysms or a neurological complication (intraventricular hemorrhage, vasospasm, and edema) showed worse output only in the last 45 seconds of the phonemic test. Patients performed comparably to control subjects on the semantic test. *Conclusions*. These results support a “diffuse damage” hypothesis of aSAH, indicated by late phase phonemic fluency impairment. Overall, the phonemic and semantic tests represent a viable, rapid clinical screening tool in the postoperative assessment of patients with aSAH.

## 1. Introduction

Subarachnoid hemorrhage secondary to aneurysmal rupture is a condition affecting approximately 1 per 10,000 persons annually in North America [1]. Aneurysmal subarachnoid hemorrhage (aSAH) is associated with a poor prognosis, having a 30-day mortality rate of 30 to 40% and high rates of impairment in day-to-day functioning. Four to 12% report decreased activities of daily living (activities relating to self-care, such as feeding, bathing, grooming, dressing, hygiene, and toileting), 44 to 93% report decreased instrumental activities of daily living (activities relating to independent living, such as the ability to use the phone and the ability to handle finances), and approximately 30% do not return to their previous occupation [2, 3]. Fifty percent of individuals who survive an aSAH are left with permanent cognitive deficits [4, 5] spanning a number of domains, including memory, language, and executive function [3]. Advances in detection and treatment have decreased rates of mortality and disability but whether these advances have changed cognitive outcomes remains unknown [6].

Gold standard tests/batteries of language and executive function, although thorough, are typically labour intensive. Often, the examiner must be trained on complex rules of administration and scoring, and expensive testing materials may be required. While the phonemic and semantic fluency tests [7] cannot replace an exhaustive battery, they are fast, easily administered, bed-side tasks which have been successfully applied to patients with aSAH and other disorders as a screening tool to assess language deficits. In a typical administration, the subject is given 1 minute to name as many words as they can that begin with a given letter (e.g., F, A, and S; phonemic fluency) or belong to a given category (e.g., animals; semantic fluency). The number of words generated and the pattern of word production, such as when the subject generates a sequence of words that are linked phonetically or semantically (i.e., clusters; [8]), are scored.

A number of studies have reported impairment on phonemic and semantic fluency in aSAH patients compared to neurologically intact controls [9–15], while two studies reported spared performance on phonemic and/or semantic fluency [16, 17]. Certain demographic and neurological factors associated with aSAH have been shown to affect cognitive outcomes and may be expected to similarly affect phonemic and semantic fluency; these include older age, fewer years of education, poorer clinical grade upon admission, anteriorly-located ruptured aneurysm, global cerebral edema, and left-sided infarctions [18]. While age and education have been shown to impact performance on phonemic and semantic fluency [19, 20], disease-related factors have not been thoroughly investigated.

Used together, the phonemic and semantic fluency tests provide a sensitive tool for the detection of executive and semantic impairments in the verbal domain. This is evidenced by the activation of left frontal and temporal regions in fMRI and PET studies in response to phonemic and semantic fluency tests [21–24] and by the patterns of cognitive impairment in patients with focal lesions in these areas [25–30]. The phonemic test is thought to rely more heavily on executive processes such as planning and strategic search, whereas the semantic test (as the name suggests) is more dependent on semantic factors such as lexical organization and retrieval. This interpretation, however, is too simplistic as both the phonemic and semantic tests engage both domains (i.e., executive and semantic). A more nuanced understanding of verbal fluency is impeded by the common practice of using a single composite score—total words generated—to interpret performance.

Investigators have begun to explore the utility of examining initial versus late phase production in patients who show impaired verbal fluency. Over the 60-second administration time, both the quantity and the quality of the words produced are known to change with time [31, 32]. Initial word generation (typically defined as the first 15–30 seconds of administration) is reliant on rapid access of salient, prototypical letter- and category-consistent words, subserved at least in part by anterior language areas in the case of phonemic fluency and cortical semantic areas for semantic fluency ([33–35]; observations in patients with cognitive complaints and Alzheimer's Disease). Investigations of individuals with real and transient cerebellar lesions and of children with attention deficit/hyperactivity disorder have also ascribed selective impairment on the first 15 seconds to a failure of automation [36–38]. With respect to late phase production, Fernaeus and Almkvist [33] support the notion of an additional mechanism that comes online at some midway point that relies on more effortful retrieval. This second phase is related to flexibility in search strategy [39], as well as a greater reliance on inherent lexico-semantic knowledge [40] and/or autobiographical information [41]. These clinical studies support cognitive and neurological differences between initial and late phase production that have not yet been investigated in the context of aSAH.

The current investigation has two goals. The first is to elucidate the phonemic and semantic fluency impairment observed in patients with aSAH through the separate analysis of initial and late phase production. If the patients with aSAH show reduced output in the first 15 seconds compared to age- and education-matched controls, the deficit may be attributed to executive dysfunction implicating frontal network changes. If reduced output is observed in the last 45 seconds, the deficit may point to more diffuse damage affecting multiple systems relating to the semantic store, effortful retrieval, self-monitoring, and sustained attention. These are higher order cognitive processes and are thus more vulnerable to disruption. The second goal is to isolate factors that are known to influence fluency and similar tasks and evaluate their relationship with initial and late phase production. We anticipate that patients treated with surgical clipping will perform worse than patients treated by endovascular coiling and healthy control subjects for the entire duration of the test due to the more damaging impact of possible microsurgical access related brain injury. Therefore, we expect that both the frontal and diffuse frontotemporal areas subserving early and later phase performance, respectively, are more compromised in clipped patients compared to the thromboembolic complications of the endovascular procedure. We will also examine how other medical factors, such as aneurysm characteristics, aneurysm-related complications, and vascular health, as well as demographic factors affect initial and late word generation.

## 2. Methods

### 2.1. Participants

Seventy-two patients with aSAH were recruited from St. Michael's Hospital, representing approximately one-third of the eligible patients seen consecutively over a 60-week period. In the vast majority of cases, withholding consent was not related to clinical factors. Patient participants met the following criteria: aSAH confirmed by computed tomography (CT), fluent English speaker, and able to participate in neurocognitive and functional testing. Exclusion criteria included traumatic brain injury beyond grade 2 concussion (based on American Neurological Association Guidelines), other brain pathology, major mental illness, substance abuse, and an estimated IQ of less than 85 (based on the National Adult Reading Test; [42]). Aneurysms were treated with endovascular coiling (*N* = 55) or surgical clipping (*N* = 17). Patients ranged from 34 to 75 years of age (*M* = 54.4; SD = 9.3) and 7 to 28 years of education (*M* = 14.6; SD = 3.5). The majority (85%) of patients were assessed at least 2 months after treatment and up to 107 months after treatment (*M* = 20.86; SD = 22.27). Twenty-five healthy controls matched for age and education were recruited for comparison. Patient characteristics are displayed in Table 1.

The project was approved by the Ethics Review Board at St. Michael's Hospital. Informed consent was obtained prior to participation.

### 2.2. Procedure

Patients were administered the phonemic and semantic fluency tests as part of a larger assessment that included self-report questionnaires and other cognitive tasks. For the phonemic fluency test, participants were instructed to orally generate as many words as they could in 60 seconds that begin with the letter “F”, while avoiding repetitions, proper nouns (e.g., “Bob” or “Boston”), or variations on the same root word (e.g., “bake” and “baking”). This procedure was repeated for letters “A” and “S”. For the semantic test, participants were asked to list as many animals as they could in 60 seconds. Responses were recorded in 15 second intervals, and scoring procedures were adapted from the work of Stuss et al. [25]. Measures of interest included number of words produced, errors (rule violations), mean cluster size (mean number of words produced consecutively that have an apparent phonemic or semantic link), and switches (shifts from one cluster to another). A subset of participants had additional neuropsychological data that included the Montreal Cognitive Assessment (MoCA; [43]; 88%), a test of global cognitive abilities; the Digit Span subtest of the Wechsler Adult Intelligence Scale ([44]; 58%), a test of working memory; and the Sustained Attention to Response Task (SART; [45]; 78%), a sustained attention task lasting approximately 20 minutes.

Additional information collected included level of English proficiency, demographics (age, education, and sex), self-report depression and anxiety symptomatology, aneurysm characteristics (location, number, and size) and aneurysm-related complications observed on CT and angiography in the hours and sometimes days following aneurysm rupture (presence of intraventricular hemorrhage (IVH), hydrocephalus, vasospasm, edema, and infarction), and neurovascular risk factors (history of hypertension, hypercholesterolemia, and smoking habit).

### 2.3. Statistical Analysis

Analyses were performed using SPSS 19.0. The phonemic and semantic fluency scores were separated into initial performance (in the first 15 seconds of the task; T15) and late phase performance (in the last 45 seconds; T45). The performance of patients with clinical risk factors purported to affect cognition was compared to that of patients without the clinical characteristics in question and to that of healthy control subjects using independent samples *t*-tests and the Mann-Whitney *U* test for nonparametric data. Effect sizes were reported as Pearson's correlation coefficients *r*. Patients treated by coiling and by clipping were analysed separately due to suspected (and confirmed) large-scale differences. For subgroup comparisons, clipped patients were omitted due to small sample size. Covariates such as age, education, depression, and various clinical factors were considered in all group comparisons. All analyses were two-tailed and considered significant if *P* < .05.

## 3. Results

### 3.1. Effect of Treatment

**Figure 1:**
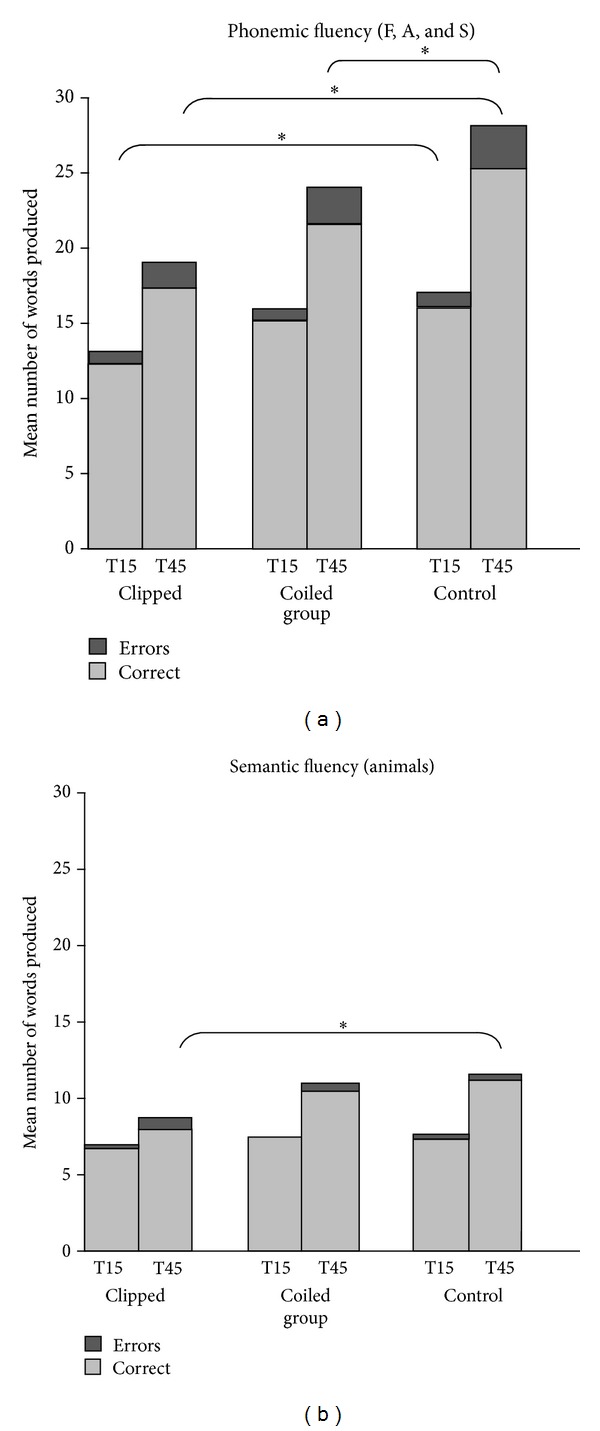
Number of words produced in the first 15 and last 45 seconds of the phonemic fluency test (a) and semantic fluency test (b). *Significantly impaired compared to healthy control subjects (*P* < .05).

Healthy control subjects were matched to patients treated by endovascular coiling and those treated by surgical clipping on age and education. The mean performance on phonemic and semantic fluency tests for all three groups by time interval is shown in Figure 1 and the performance of clinical groups and subgroups distinguished by aneurysm location, number, and complications compared with healthy control participants is presented in Table 2.

Patients who have undergone coiling produced fewer words compared to control subjects in the last 45 sec. only (*t*(78) = − 2.10, *P* = .039, and *r* = .23). Their error, switch, and cluster scores were comparable to those of controls. On the semantic test, coiled patients produced more errors than controls in the first 15 sec. (*U* = 605.00,  *P* = .009, and *r* = .29).

Patients who have undergone clipping produced fewer correct words on the phonemic test in both the first 15 (*t*(40) = − 3.56, *P* = .001, and *r* = .49) and the last 45 sec. (*t*(40) = − 3.87, *P* < .001, and *r* = .52) compared to control subjects. Controls also made more switches than clipped patients (T15: *t*(40) = − 2.15, *P* = .038, *r* = .32; T45: *t*(40) = − 2.30, *P* = .027, and *r* = .34), though the two groups did not differ on error production or mean cluster size. On the semantic fluency test, control subjects produced more correct words and more switches compared to clipped patients in the last 45 sec. (resp., *t*(40) = − 2.48, *P* = .017, and *r* = .37; *U* = 111.00, *P* = .008, and *r* = .41).

**Figure 2:**
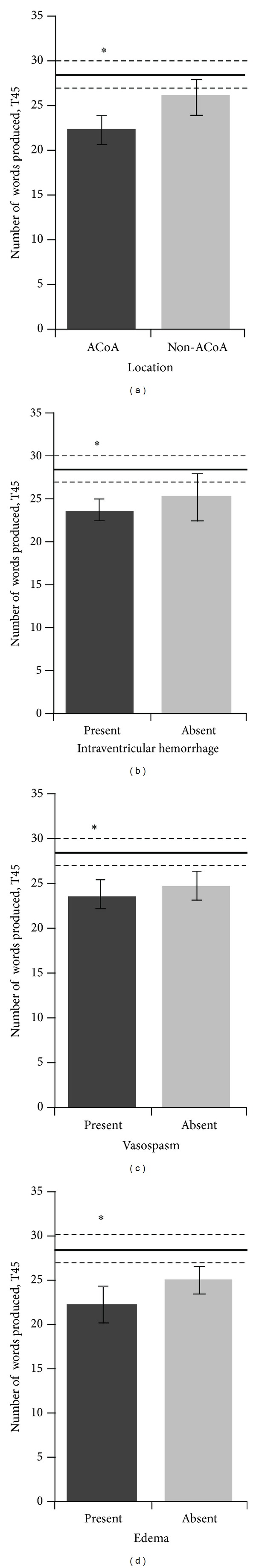
Number of words produced in the last 45 seconds of the phonemic fluency test by subgroups of coiled patients. Groups represented by the dark bars are those that were uniquely impaired in the last 45 seconds compared to healthy control subjects (*P* < .05). The bold horizontal line represents mean production in the control group; the dashed lines represent SEM.

Coiled patients performed better than their clipped counterparts on the phonemic test, producing more words in the first 15 sec. (*U* = 282.50, *P* = .014, and *r* = .29) and the last 45 sec. (*t*(70) = 2.11, *P* = .039, and *r* = .24). In the semantic test, they made fewer errors in the first 15 sec. (*U* = 412.50, *P* = .010, and *r* = .30) and produced more correct words and more switches in the last 45 (*t*(70) = 2.44, *P* = .017, and *r* = .28; *U* = 268.00, *P* = .008, and *r* = .31). It is worth noting that the clipped group had fewer patients with Anterior Communicating Artery (ACoA) aneurysms than the coiled group (*χ* ^2^(1, *N* = 62) = 5.89, *P* = .015). However, this does not explain their impaired performance since the presence of an ACoA aneurysm appears to be an indicator of poor performance (see effect of location in Figure 2).

### 3.2. Demographic Factors

Clipped patients are not included in subgroup analyses due to small sample size. Statistics from this point relate to coiled patients only. None of the demographic factors (age, education, and sex) were correlated with phonemic or semantic fluency variables.

### 3.3. Aneurysm Characteristics

- Location: on the phonemic test, patients with ruptured ACoA aneurysms produced fewer correct words in the last 45 sec. than the healthy control subjects (*t*(49) = − 2.81, *P* = .007, and *r* = .37) but performed comparably to patients with non-ACoA aneurysms. On the semantic test, the ACoA group was not significantly different from the non-ACoA group or healthy controls.
- Number: on the phonemic test, coiled patients with multiple aneurysms produced fewer words across the 60 sec. compared to control subjects (T15: *t*(39) = − 2.45, *P* = .019, and *r* = .37; T45: *t*(39) = − 2.14, *P* = .039, and *r* = .32) but performed comparably to patients with single aneurysms. On the semantic test, neither patients with single aneurysms nor patients with multiple aneurysms showed impairment compared to healthy controls.
- Size: for patients with singular aneurysms, the size of the aneurysm was categorized as small (<7 mm), medium (7–12 mm), or large (13–24 mm; [46]). Twenty-one (38%) of the coiled patients had a small aneurysm, 16 (29%) had a medium aneurysm, and 1 (2%) had a large aneurysm. Size category was found to moderately correlate with mean cluster size over the 60 sec. on the phonemic test (larger aneurysms were associated with larger cluster sizes; *r*_*s*_(36) = .35, *P* = .033) and with error production, specifically in the last 45 sec., on the semantic test (*r*_*s*_(36) = .35, *P* = .029).

### 3.4. Complications

Compared to healthy control subjects, patients with IVH, vasospasm, or edema produced fewer words on the phonemic fluency test in the last 45 sec. only (resp., *t*(64) = − 2.21, *P* = .031, and *r* = .27; *t*(51) = − 2.03, *P* = .043, and *r* = .27; *t*(39) = − 2.47, *P* = .013, and *r* = .32). Additionally, patients with and without vasospasm differed on clusters in the first 15 sec. and error frequency in the last 45 sec. (*U* = 206.00, *P* = .006, and *r* = .37; *U* = 203.00, *P* = .004, and *r* = .39). On the semantic fluency test, no effects of complications were observed.

### 3.5. Neurovascular Risk Factors

A past medical history positive for hypertension, hypercholesterolemia, or a smoking habit did not impact phonemic or semantic fluency performance.

### 3.6. Neuropsychological Tests

MoCA, Digit Span, and SART were correlated with the number of words correctly produced in the first 15 sec and last 45 sec in both the phonemic and semantic fluency tests to further validate our results. The MoCA, a measure of global cognitive ability, was correlated with production in the first 15 (*r*_*s*_(63) = .39, *P* = .002) and last 45 sec. (*r*_*s*_(63) = .37, *P* = .003) of the phonemic fluency test. Performance on Digit Span Forwards, reciting a list of numbers in the order they were presented, was selectively correlated with correct production in the first 15 sec. (*r*_*s*_(42) = .35, *P* = .025) while the Backward Condition, reciting a list of numbers in the reverse order of their presentation, was correlated with both correct production in the first 15 (*r*_*s*_(42) = .40, *P* = .009) and last 45 sec. (*r*_*s*_(42) = .32, *P* = .039). Finally, errors of omission (failing to respond to the target stimulus in a sequence) and errors of commission (responding to nontarget stimuli) on the SART were not significantly correlated with correct production in the first 15 or last 45 sec.

With regard to the semantic fluency test, SART omission errors correlated with correct production in the last 45 sec. (*r*_*s*_(56) = − .40, *P* = .007). No other correlations with neuropsychological variables were observed.

## 4. Discussion

Patients with aSAH were selectively impaired on the last 45 seconds of the phonemic fluency test but were unaffected in the initial 15 seconds. This pattern was observed in coiled patients as a whole, as well as in subgroups distinguished by the presence of an ACoA aneurysm, IVH, vasospasm, and edema (see Figure 2). While certain groups showed a deficit across the entire task (patients with a clipped aneurysm and patients with multiple aneurysms), none showed a selective impairment in the first 15 seconds when compared to controls. In the present sample, the semantic fluency test was relatively insensitive to overall aSAH pathology and specific clinical characteristics.

Performance in the first 15 seconds of word generation, thought to reflect primarily executive functioning, did not differ between coiled aSAH and control participants. This finding may appear discordant with previous literature which has documented executive deficits following aSAH. However, executive function is a broad term referring to a variety of higher order cognitive processes. For this reason, prevalence estimates of executive dysfunction in aSAH are highly variable (3–76%; [3]). Certain subdomains are reliably degraded in aSAH survivors, including inhibition (commonly assessed by the Stroop task), problem-solving (assessed by the Tower of London), and cognitive flexibility (assessed by the Wisconsin Card Sorting Test) [5, 11, 47, 48]. Other processes such as task initiation or automation, which are thought to be the foundation of initial verbal fluency performance, have not been investigated in aSAH. The results suggest that the neural correlates of task initiation such as left medial frontal areas [25] are preserved in aSAH and in the coiling procedure but are more likely to be disrupted in the clipping procedure and in the presence of multiple aneurysms.

A possible explanation for the disruption of late phase production may lie in the neural correlates of production. It is well documented that the semantic fluency test is subserved by regions of the temporal lobe and that phonemic fluency is dependent on regions of the frontal lobe [21–24, 26, 28, 29]. These regions are necessary but not sufficient for performance. Reitan and Wolfson [49] point out that the description of phonemic fluency as a “frontal” task is accurate because the frontal lobe involvement in phonemic fluency is undeniable, but at the same time, it is misleading since one may wrongfully assume that it is a uniquely frontal lobe task. A review by Alvarez and Emory [50] demonstrates the good sensitivity but poor specificity of phonemic fluency performance to frontal lesions. That is, patients with nonfrontal lesions may also show phonemic fluency impairment [11, 25, 36, 51]. The contributions of frontal and nonfrontal regions to fluency performance have not been sufficiently defined. Additionally, previous studies failed to consider the temporal profile of word generation and the unique neural correlates of initial versus late phase production.

There is evidence to suggest that initial phonemic fluency production is localized to regions of the frontal lobe [52]. Fernaeus et al. [34] showed that initial phonemic fluency performance was selectively correlated with anterior white matter lesions in patients with memory complaints. Conversely, they found no relationship between late production and anterior or nonanterior regions of white matter lesions. This result suggests that rather than being localized to one region, late phase production may be related to a distributed neural network comprised of both frontal and temporal language areas and their interactive pathways. While initiating processes such as planning and strategy selection (frontal executive functions) are essential in the first 15 seconds, these give way to the effortful search for alternative strategies and a deeper excavation of the semantic store [53]. In addition, sustained attention and self-monitoring become more cumbersome with time. We observed a correlation between production in the last 45 seconds and the Digit Span Backwards but not the Forward Condition, suggesting a greater emphasis of working memory demands in late phase production. The neural substrates of these late phase, executive, and semantic processes are necessarily distributed. This would explain why patients with IVH, vasospasm, or edema—indicators of diffuse cerebral damage—showed selective impairment in the last 45 seconds of the task.

Given the diffuse nature of aSAH, the distributed network responsible for sustained production is highly vulnerable to disruption at one or more foci, resulting in impaired performance. Prior to aneurysm rupture, risk factors such as hypertension, hypercholesterolemia, and smoking can cause chronic and widespread white matter disease [54, 55]. Aneurysm rupture triggers an increase in intracranial pressure and a decrease in cerebral blood flow which can last several minutes [56]. The net effect can be global ischemic injury, affecting multiple pathways and predictive of later morbidity [57]. Complications following aneurysm rupture (such as IVH, vasospasm, and edema) as well as complications following treatment (such as microthromboemboli) cause further damage. Bendel et al. [58] observed ventricular and sulcal enlargement in chronic aSAH patients suggestive of stable, diffuse brain atrophy. Cognitive impairment in at least one domain tested (general intellectual functioning, memory, language, and executive functions) was associated with greater diffuse atrophy. If the association fibres linking the frontal and temporal language areas were affected, impaired sustained production on the phonemic fluency test might be an expected consequence. This is consistent with our finding that ACoA aneurysms negatively impact late production since the ACoA territory is largely posterior frontal/medial temporal. Future investigations of the unique neuroanatomical and white matter correlates of initial versus late phase production in a larger population are needed to validate this claim and the use of verbal fluency tasks in the assessment of patients with aSAH.

Patients with clipped aneurysms exhibited impairment in the last 45 seconds of the semantic fluency and across the entire 60 seconds of administration in the phonemic fluency when compared to controls. This was consistent with other studies that found clipped patients to be more impaired on phonemic and semantic fluency than coiled patients, although the temporal profile has never been investigated [13, 14, 16, 17]. This is not surprising given the invasive nature of the surgical method that, unlike endovascular coiling, requires craniotomy, brain tissue retraction, and direct manipulation of arteries which may damage perforating branches and consequently reduce blood flow [14, 59]. Moreover, vasospasm, ischemic deficits, and encephalomalacia may be consequences of the clipping procedure [13, 60]. The disruptive effects of surgical intervention, compounded by the diffuse injury associated with aSAH, appear to contribute to a broader, more severe deficit in phonemic fluency than is seen in aSAH patients having undergone endovascular coiling. It should be noted that aneurysm location and morphology are crucial in the selection of treatment type, thus introducing a bias.

Consistent with two previous studies [16, 17], semantic fluency performance was intact in our coiled patients. Language is organized semantically in the brain such that retrieval of one word causes a cascade of activation, priming the retrieval of semantically—and not phonemically—similar words [21, 61]. A semantic cue prescribes the subject's search parameters, placing less emphasis on executive involvement in word fluency. Conversely, a phonemic cue presents a large pool of acceptable responses that necessitates strategic search and cognitive flexibility. Thus the phonemic fluency test requires more mental effort than the semantic test. This disparity is demonstrated in neurologically intact subjects, who show better performance on semantic than phonemic fluency [20, 39, 62, 63]. Since a handful of studies have demonstrated semantic fluency deficits in aSAH [9–13], another possible explanation is that the present sample has less profound impairment than in previous investigations, potentially limiting the generalizability of our conclusions.

aSAH is a clinical population associated with neurological deficits that range from subtle to severe, which makes the utility of an objective quick assessment tool highly invaluable. The division of fluency tests into initial and late phase production may provide a snapshot of impairment that aids in the identification of specific cognitive deficits in aSAH. While we are not suggesting that these tests can replace the exhaustive, validated neuropsychological batteries currently in use, the semantic and phonemic fluency tests are rapid and sensitive, bed-side screening tools for language, executive, and semantic deficits. With the addition of temporal information, these tests can be used to identify candidates for more detailed neuropsychological testing.

## Figures and Tables

**Table 1:** Participant demographics and aneurysm characteristics.

Treatment	aSAH		Control
	Coil	Clip	·
Sample size	55	17	25
Age, in years (M ± SD)	54.13 ± 8.80	55.25 ± 11.01	56.56 ± 13.42
Education, in years (M ± SD)	14.63 ± 3.65	14.44 ± 3.22	15.48 ± 1.81
Sex (M, F)	20, 35	5, 12	13, 12
Location (ACoA, non-ACoA)*	26, 23	2, 11	·
Number (single, multiple)	39, 16	9, 8	·
Size (small, medium, large)	21, 16, 1	5, 2, 1	·

*Missing values represent patients with multiple aneurysms with at least 1 ACoA and 1 non-ACoA aneurysm.

**Table 2:** Initial (T15) and late phase (T45) word production compared to healthy controls.

Group	*N*	T15			T45		
		M	SD	*P*	M	SD	*P*
Control	25	17.08	3.32		28.36	7.76	
Clip	17	13.18	3.73	0.001	19.24	7.12	<0.001
Coil	55	15.91	3.74	ns	24.11	8.66	0.039
Location							
ACoA	26	15.77	3.63	ns	22.23	7.83	0.007
Number							
Multiple	16	14.38	3.67	0.019	22.81	8.62	0.039
Complications							
IVH	41	15.56	3.72	ns	23.76	8.47	0.031
Hydrocephalus	27	16.26	2.88	ns	24.96	7.48	ns
Vasospasm	28	15.39	3.98	ns	23.5	9.46	0.043
Edema	16	14.94	3.99	ns	22.19	7.93	0.013
Infarct	22	16.27	3.74	ns	24.77	9.17	ns

*N*: sample size, M: mean, SD: standard deviation, *P*: significance level for comparison with healthy control subjects, ns: nonsignificant; ACoA: anterior communicating artery, IVH: intraventricular haemorrhage.
